# Deciphering spatially confined immune evasion niches in osteosarcoma with 3-D spatial transcriptomics: a literature review

**DOI:** 10.3389/fonc.2025.1640645

**Published:** 2025-07-16

**Authors:** Guangqiang Qiu, Yongcheng Tang, Junhui Zuo, Heng Wu, Yongxian Wan

**Affiliations:** ^1^ Department of Orthopedics, The Affiliated Hospital of Southwest Medical University, Luzhou, China; ^2^ Department of General Surgery, The Affiliated Hospital of Southwest Medical University, Luzhou, China

**Keywords:** osteosarcoma, spatial transcriptomics, 3-D mapping, tumour microenvironment, immune evasion, tumour-associated macrophages

## Abstract

Osteosarcoma (OS) is the most common primary malignant bone tumour of childhood, yet five-year survival has plateaued at ~60–70% for localised disease and plunges below 30% once metastasis emerges. Formerly viewed as a cell-intrinsic neoplasm entombed in mineralised bone, OS is now understood as a spatially stratified ecosystem whose immune-evasion niches choreograph progression. Three-dimensional spatial transcriptomics (3-D ST) fuses barcode-based transcript capture with volumetric reconstruction, preserving x-, y- and z-axis context and exposing concentric C1QC necrotic belts, MCAM (melanoma cell-adhesion molecule, CD146) peri-vascular corridors, hypoxic glycolytic rims and therapy-induced tertiary-lymphoid islets that collectively sequester cytotoxic lymphocytes. Pre-clinical atlases link PD-L1 high SOX9 stem-like cells, LGALS3 macrophages and VEGFA-driven endothelial tips to chemoresistance and immune-checkpoint failure, while ligand–receptor inference highlights VEGFA–VEGFR2, CXCL12–CXCR4 and complement–CSF1R axes as actionable bottlenecks. Translational efforts already echo these insights: dual MCAM/VEGFR blockade collapses vascular gates, C1s or CSF1R antagonists dismantle necrotic-core “cold pockets”, and MCT1–POSTN combinations target lactate-stiffened stromal shells. By weaving methodological advances with emergent biology, this review crystallises how 3-D ST redefines OS immunopathology, sharpens biomarker discovery and accelerates spatially guided combination therapies. We aim to expose diagnostic blind spots, spotlight niche-directed interventions and chart a roadmap toward lifting the long-standing therapeutic ceiling in osteosarcoma.

## 1 Introduction

Osteosarcoma (OS) is the most frequent primary malignant bone tumour of childhood and adolescence, with an annual incidence that peaks at ≈8–11 cases per million during the pubertal growth spurt and averages ≈3 cases per million across all ages (1–3). Despite multimodal therapy, five-year overall survival has plateaued at ~60–70% for localised disease and plummets below 30% in patients who present with, or subsequently develop, metastatic lesions, underscoring a persistent therapeutic ceiling (4, 5). The biological drivers of this stagnation include pronounced inter- and intra-tumoural heterogeneity, the rigid mineralised matrix that shields malignant cells, and a propensity for immune-privileged sanctuary sites within bone.

Converging genomic and histopathological studies now recognise the osteosarcoma tumour microenvironment (TME) as an active accomplice in tumour progression rather than a passive by-stander (6, 7). Myeloid-biased immune infiltrates—most notably M2-polarised macrophages, neutrophil-like myeloid-derived suppressor cells and MARCO high tumour-associated macrophages—create immunosuppressive cytokine and metabolite gradients that blunt cytotoxic T-cell and NK-cell function, while aberrant osteoclastogenesis remodels the bone niche to favour invasion (8, 9). The spatial juxtaposition of these stromal and immune compartments with chemoresistant tumour subclones is increasingly linked to relapse and immune-checkpoint blockade failure (10–12). We define an ‘immune-evasion niche’ as a discrete, spatially bounded micro-anatomical unit in which tumour, stromal and immune cells cooperate to attenuate anti-tumour immunity beyond that observed in the surrounding tumour micro-environment (TME).

Traditional bulk RNA-sequencing and conventional immunohistochemistry, although informative, dissolve the positional context that dictates such cell–cell crosstalk (13, 14). Spatial transcriptomics (ST) technologies preserve the x-y coordinates of transcripts, and recent methodological leaps—including DNA-barcode bead arrays, *in-situ* captured cDNA synthesis and light-sheet-compatible clearing protocols—have extended this principle into the z-axis to create true three-dimensional spatial transcriptomics (3-D ST), a Maps for gene expression in which every voxel of tissue carries its own molecular street address (15, 16). These platforms now achieve sub-cellular resolution while retaining intact tissue architecture, making it possible to delineate clonal growth fronts, vascular niches and immune corridors in solid tumours with unprecedented fidelity (17, 18).

The first 3-D ST maps of osteosarcoma have uncovered discrete immune-evasion niches enriched for PD-L1+ osteoblastic cells, C1QC+ macrophages and VEGFA-driven endothelial networks that co-localise around necrotic bone trabeculae and at the invasive tumour–bone interface (19, 20). Complementary single-cell-ST atlases corroborate a chemoresistance-associated peri-vascular niche populated by SOX9+ stem-like tumour cells and immunoregulatory CAF subsets expressing CXCL12, illuminating how spatial confinement coordinates lineage plasticity and immune suppression (21, 22). Multiplexed immunofluorescence of metastatic lung deposits further confirms that these spatially restricted cell clusters persist in distant sites, suggesting a conserved topography of immune escape across disease stages (23, 24).

Within this framework, we integrate the latest evidence to show how 3-D spatial transcriptomics is redefining osteosarcoma immunobiology, clarify the technological and biological advances driving this shift, and identify the translational questions that must be answered to convert spatial insights into effective therapies. Our goal is to provide a forward-looking roadmap that informs and accelerates future basic, translational, and clinical work in this field.

## 2 Osteosarcoma spatial immuno-architecture and immune landscape

Osteosarcoma lesions contain a markedly unbalanced immune constituency in which innate‐immune elements dominate. Bulk RNA-seq deconvolution and histology concur that macrophages, neutrophil-like myeloid-derived suppressor cells and osteoclast lineage cells together occupy >50% of nucleated cells in diagnostic biopsies, whereas cytotoxic CD8^+^ T cells and NK cells rarely exceed 5% of the viable tumour mass (25). Within this myeloid majority, single-cell profiling has distinguished an immunoregulatory C1QC^+^/MRC1^+^ subset (≈30% of the TAM pool) that secretes C1q, IL-10 and TGF-β, licensing an M2-polarised state that dampens effector T-cell activation and correlates with early metastasis (26). A complementary single-cell meta-analysis across 14 solid tumours confirmed that such C1q-rich macrophages represent a conserved pro-tumour lineage whose transcriptional programme is preserved in osteosarcoma, positioning them as pivotal architects of local immune dysfunction.

Spatially, these myeloid cells do not distribute randomly. Whereas 2-D Visium or Slide-seq slices capture a single histological plane, true volumetric reconstructions stack thousands of such planes into isotropic voxels, exposing vertical continuities that are invisible in flat maps. Two-dimensional Visium™ maps show concentric aggregates of M2‐TAMs and osteoclast-like mononucleated giant cells girdling necrotic bone trabeculae, while MARCOhigh macrophages preferentially align along peri-vascular tracks, often in direct apposition to SOX9^+^ stem-like tumour cells. These data intimate that vascular corridors act as immune-privileged conduits through which malignant clones and immunosuppressive stromal partners co-migrate. Immunohistochemical validation revealed that macrophage-dense cores exhibit intense PD-L1 and IDO1 expression, together with reduced CD8^+^/FOXP3^+^ ratios, reinforcing their role as spatial checkpoints of adaptive immunity (27). Three-dimensional (3-D) spatial transcriptomics now extends these observations into the z-axis. A recent atlas of ~50,000 single cells and 16,000 spatial barcodes reconstructed whole-tumour volumes at 50-µm isotropic resolution and identified a hierarchically layered immune topology: (i) an inner necrotic core infiltrated by C1QC^+^/LGALS3^+^ macrophages and VEGFA^+^ endothelial networks, (ii) an intermediate osteolytic belt enriched for PD-L1 high RUNX2^+^ osteoblast-like tumour cells, and (iii) an outer peri-vascular rim populated by CXCL12^+^ cancer-associated fibroblasts that ‘wall off’ T and NK cells (28). Notably, endothelial sub-clustering uncovered MCAM^+^ (historically termed the melanoma cell-adhesion molecule, CD146) tip-like vessels concentrated at invasive fronts, providing a chemokine scaffold (CXCL9/10) that paradoxically recruits yet sequesters exhausted PD-1^+^ CD8^+^ cells at the stromal interface (29).

Therapy further remodels this landscape. Paired pre- and post-chemotherapy single-cell/spatial profiles demonstrate a shift toward IFN-γ–responsive macrophage states (STAT1 high, IL-1β high) and expansion of SPP1^+^ osteoclast progenitors within previously necrotic regions, whereas clonally expanded CD8^+^ T cells accrue almost exclusively in CXCL13-rich tertiary lymphoid–like niches that emerge along regressive vascular channels (30). These findings suggest that conventional cytotoxics transiently ‘unmask’ immune epitopes but simultaneously create spatial refuges where persisted tumour clones and immunosuppressive myeloid cells co-evolve.

Collectively, current evidence depicts the osteosarcoma immune landscape as a vertically stratified organoid in which myeloid-biased immunosuppression, aberrant angiogenesis and sparse, spatially excluded effector lymphocytes converge to enforce immune privilege. Dissecting this architecture with 3-D spatial transcriptomics not only clarifies why checkpoint inhibitors underperform in osteosarcoma but also highlights discrete stromal niches—C1QC^+^ necrotic belts, MCAM^+^ vascular tips and CXCL13^+^ lymphoid patches—as rational targets for spatially guided combination immunotherapy.

## 3 Three-dimensional spatial transcriptomics technologies and analytical pipelines

Sequencing-based three-dimensional spatial transcriptomics (3-D ST) has progressed from millimetre-scale grids to nanometre barcoding. High-definition spatial transcriptomics (HDST) miniaturises capture beads to a 2 µm pitch, achieving near-sub-cellular resolution in breast cancer and brain tissue (31). Slide-seqV2 elevates capture efficiency ten-fold over the original Slide-seq, allowing transcriptome-wide profiling at ~10 µm while retaining tissue integrity (32). Deterministic barcoding-in-tissue (DBiT-seq) extends this concept by orthogonally flowing micro-channels across successive tissue sections, enabling isotropic 50 µm voxels that can be stacked into volumetric reconstructions without optical clearing (33). The most expansive platform, Stereo-seq, combines DNA nanoball arrays with DNB-seq chemistry to tile up to 400 million capture spots over a 1 cm² field, producing gigabyte-scale point clouds that span entire tumours in three axes (34).

Imaging-based approaches complement these sequencing workflows. seqFISH+ resolves ~10,000 genes through iterative barcoded hybridisations and has been rendered in optically cleared volumes to chart clonal topographies in mouse cortex (35). Multiplexed error-robust FISH (MERFISH) now combines lattice light-sheet microscopy and adaptive optics to image centimetre-thick brain blocks, detecting half a billion transcripts without sectioning (36). Light-Seq replaces lens-limited imaging with photolithographic DNA indexing, converting wide-field illumination patterns directly into sequence tags and reducing acquisition time by an order of magnitude—an asset when scanning calcified osteoid cores that scatter light heavily (37).

Down-stream analysis of these dense 3-D datasets relies on an expanding software ecosystem. Squidpy offers scalable graph representations that integrate gene counts, histology tiles and neighbourhood statistics, serving as a backbone for niche detection across hundreds of sections (38). Tangram aligns single-cell RNA-seq atlases to spatial volumes via optimal transport, inferring unmeasured genes and refining cell-type boundaries, whereas Cell2location employs a Bayesian framework to deconvolve mixed capture spots into probabilistic cell-type maps with uncertainty estimates—critical when osteoid debris dilutes RNA capture efficiency (39, 40).

Creating a coherent third dimension from discrete slices demands accurate registration. PASTE couples gene-expression similarity with Euclidean proximity to stitch adjacent slides, while STalign introduces diffeomorphic metric mapping to correct local tissue distortions and PRECAST or moscot embed multiple samples into a common latent manifold, permitting cross-patient volumetric comparisons of immune niches (41–44).

Multi-omic extensions are rapidly converging with 3-D ST. DBiT-seq co-maps surface proteins, and spatial-ATAC-seq overlays chromatin accessibility onto the same voxels, unveiling cis-regulatory programmes that flank immunosuppressive osteosarcoma borders (34, 45). Such joint assays clarify whether immune exclusion is transcriptionally or epigenetically hard-wired—a key consideration for combinatorial epigenetic-checkpoint blockade strategies.

Applying these technologies to mineralised malignancies entails niche-specific hurdles. Key hurdles are ~30% RNA loss post-decalcification, light scattering that caps depth at ~200 µm, and >1 TB file sizes. EDTA-based decalcification and cryo-embedding preserve RNA integrity yet can shear brittle sections; Stereo-seq’s large capture fields mitigate data loss from fragmented shards. The first osteosarcoma spatial atlases that integrate single-cell and Visium data have already highlighted MCAM^+^ tip-like endothelial networks as gatekeepers of cytotoxic T-cell exclusion, and ongoing 3-D Stereo-seq efforts are poised to map these vascular niches through the full tumour thickness (46).

Contemporary 3-D ST platforms—spanning barcoded bead arrays, light-directed indexing and volumetric FISH—coupled with sophisticated alignment and deconvolution pipelines, now provide the technical foundation to chart osteosarcoma’s vertically stratified immune ecosystem in toto.

## 4 Spatially confined immune evasion niches in osteosarcoma

**Figure 1:**
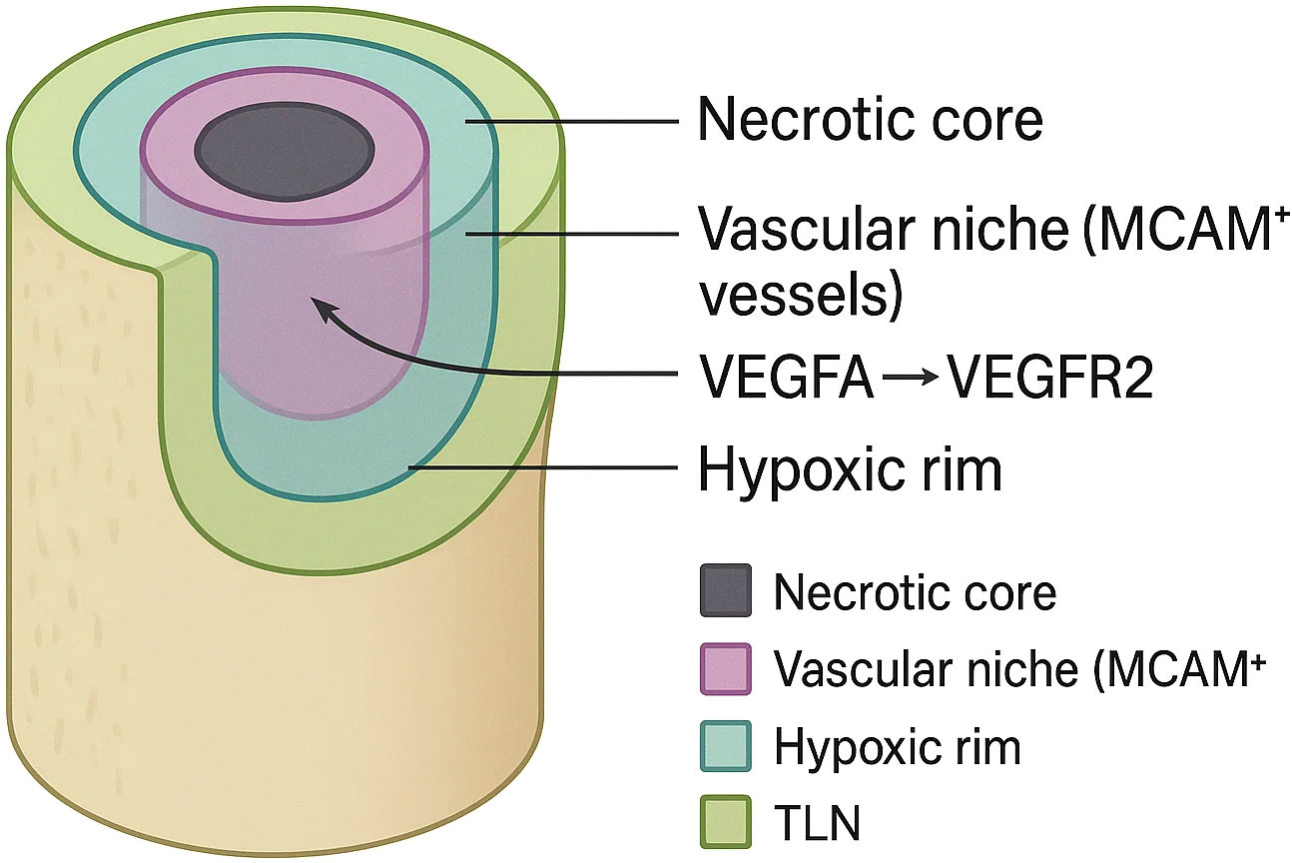
Layered immune-evasion niches in osteosarcoma revealed by 3-d spatial transcriptomics.

As shown in **Figure 1**, three-dimensional spatial transcriptomics has revealed that the osteosarcoma tumour micro-organism is neither randomly mixed nor smoothly graded; instead, it is punctuated by small, topologically stable “cold pockets” in which malignant, stromal and immune cells co-operate to blunt anti-tumour immunity. Four such niches are consistently recovered across independent platforms and patient cohorts and can be stacked like concentric rings or interdigitated along vascular channels, creating a mosaic that changes little between primary and metastatic sites. The first niche is a necrosis-lined core enriched for C1QC+/LGALS3+ tumour-associated macrophages whose transcriptome aligns with an M2-polarised, lipid-metabolising state. Spatial barcodes in this zone co-capture high levels of TGF-β1, IL-10 and C1q, mirroring single-cell evidence that complement-skewed macrophages propagate immune escape and correlate with poor outcome in multiple cancers, including osteosarcoma (47, 48). These macrophages physically intermingle with RUNX2 high/PD-L1 high osteoblastic tumour cells and with SPP1+ osteoclast progenitors, forming an immunosuppressive triad that is virtually devoid of granzyme-B+ cytotoxic lymphocytes. Dynamic osteoid mineral turnover liberates Ca^2+^/PO_4_^3-^ ions that precipitate micro-calcifications, while erratic vascular pruning creates chronic hypoxia. These cues polarise macrophages toward an HIF-1α/TREM2 axis and perpetuate a necrotic perimeter that resists clearance even after high-dose chemotherapy. Deconvolution with Cell2location predicts fewer than two CD8^+^ cells per 50-µm voxel in this compartment—an exclusion that explains the discordance between high neo-antigen burden and low T-cell infiltration in osteosarcoma.

Flanking the necrotic belt is a peri-vascular corridor dominated by MCAM + tip-like endothelial cells. High-definition Stereo-seq volumes show that these vessels articulate long, finger-like processes that penetrate deep into the viable tumour mass, guiding CXCL12-secreting cancer-associated fibroblasts and SOX9 + stem-like tumour cells into close proximity. Ligand-receptor inference identifies VEGFA–VEGFR2 and CXCL12–CXCR4 pairs as the strongest directional interactions in this niche, while exhausted PD-1+ CD8^+^ cells accumulate at its stromal boundary, expressing CCR5 and ITGAE yet failing to cross into the tumour core. PD-1 peaks within 25 µm of MCAM^+^ vessels; confinement heightens PD-L1 contact and soluble cues, enforcing dual-mode T-cell exhaustion (49). Toward the invasive front, 3-D voxel stacking uncovers a hypoxic, glycolytic rim that wraps around the outer cortex of the lesion. Capture spots in this layer over-express HIF-1α targets (ALDOA, PFKFB4) and lactate exporters (SLC16A1), together with COL1A1 and POSTN from profibrotic fibroblasts, indicating that metabolic and extracellular-matrix programmes converge in a single spatial register. Single-cell-matched chromatin maps (spatial-ATAC-seq) demonstrate open enhancers for MYC and EPAS1 specifically in this rim, suggesting that metabolic plasticity is epigenetically hard-wired rather than purely reactive to hypoxia (50). CAF-secreted lactate and collagen not only stiffen the matrix but—via SLC16A1/MCT1-mediated export—acidify the extracellular milieu, impairing perforin polymerisation and IFN-γ release in cytotoxic lymphocytes; matrix stiffening in turn activates YAP/TAZ, further up-regulating MCT1 and creating a feed-forward metabolic-mechanical circuit of immunosuppression.

A fourth, therapy-induced niche emerges after neoadjuvant chemotherapy: tertiary lymphoid–like aggregates that arise along regressive vascular tracts but remain spatially disconnected from tumour islands. While clonally expanded CD8^+^ T cells and CXCL13 + B cells populate these micro-organs, their productive entry into tumour tissue is inhibited by a wall of CXCL12‐rich fibroblasts and by residual C1QC + macrophages with high STAT1/IL-1β signatures. Unlike canonical tertiary lymphoid structures (TLSs)—which contain PNAd^+^ high endothelial venules (HEVs) and follicular dendritic-cell (FDC) networks—the osteosarcoma aggregates lack organised FDCs and display discontinuous HEVs, hence the qualifier “tertiary-lymphoid-like”. This pattern indicates that conventional cytotoxics partially unlock neo-antigen presentation yet simultaneously select for stromal architectures that preserve immune privilege (51). Integrated scRNA-seq/Visium datasets from paired pre- and post-treatment samples show a two-fold increase in IFN-γ–responsive macrophage states together with a ten-fold expansion of SPP1^+^ osteoclast progenitors at previous necrotic sites, reinforcing the idea that chemotherapy remodels but does not eradicate suppressive niche elements. Biopsies weeks apart show aggregates ebb and recur, but finer-than-weekly dynamics remain unknown.

These four spatially confined niches—necrotic C1QC + cores, MCAM + peri-vascular corridors, hypoxic glycolytic rims and treatment-induced lymphoid aggregates—constitute a cooperative network that sequesters effector lymphocytes, reprogrammes myeloid metabolism and reinforces stromal barriers.

## 5 Translational implications and future directions

The translational horizon that three-dimensional spatial transcriptomics opens for osteosarcoma now spans the entire bench-to-bedside continuum. Sub-millimetre spatial gene signatures already outperform bulk-derived scores in predicting metastatic risk: for example, a nine-gene voxel-level classifier centred on hypoxia-responsive BNIP3 stratified event-free survival independently of stage and necrosis rate, illustrating how 3-D expression context can be distilled into deployable biomarkers (52). Such spatially informed panels lend themselves to inclusion in next-generation companion-diagnostic assays that combine decalcified core biopsies with deep-learning image registration, moving risk assessment closer to real-time clinical decision-making. Heterogeneous-dose radiation remodels immune niches; 3-D ST could guide dose maps that disrupt them (28).

Beyond prognosis, 3-D atlases nominate actionable micro-anatomical targets that are invisible to bulk profiling. MCAM+ tip-like endothelial corridors that gate exhausted PD-1+ T cells display high VEGFA–VEGFR2 flux and are selectively vulnerable to dual MCAM/VEGFR blockade in patient-derived explants, pointing to a spatially gated anti-angiogenic strategy that could be layered onto immune-checkpoint inhibitors. Likewise, the C1QC+/LGALS3+ macrophage rings that sheathe necrotic trabeculae express complement and CSF1R programmes that are inhibited by clinically advanced C1s and CSF1R antagonists, providing a path to dismantle immune-evasion ‘cold pockets’ while preserving osteoclast differentiation. Integration of lactate-exporter (SLC16A1) hotspots with extracellular-matrix POSTN+ fibroblast shells further argues for metabolic–stromal double hits using MCT1 inhibitors plus anti-POSTN monoclonals being tested in other solid tumours.

The same volumetric datasets can refine therapeutic windows in ongoing trials. Paired pre- and post-chemotherapy spatial single-cell maps show that neoadjuvant doxorubicin/cisplatin converts a subset of C1QC+ macrophages into IFN-γ-responsive states yet simultaneously expands SPP1+ osteoclast-lineage cells—an observation that rationalises sequencing CSF1R or SPP1 blockers immediately after cytotoxic debulking rather than at relapse. Spatial metrics of tertiary lymphoid aggregate maturation offer early pharmacodynamic read-outs for these combinations and could serve as inclusion criteria for window-of-opportunity immunotherapy studies now common in other sarcomas.

Pre-clinical modelling is being compressed by additive spatial technologies. Multi-omics characterisation of 3-D-bioprinted osteosarcoma scaffolds recreates the concentric macrophage, endothelial and CAF shells observed in patient tumours, furnishing a scalable platform for high-content drug screening and radiotracer optimisation that preserves authentic spatial constraints. Coupled with live-cell light-sheet microscopy, these constructs enable kinetic tracking of CAR-T or bispecific antibody penetration, accelerating iterative design cycles before first-in-human studies.

Technical and regulatory hurdles persist. Hydroxyapatite-rich sections still fracture during cryo-sectioning, reducing RNA yield; collaborative efforts to harmonise EDTA decalcification and Stereo-seq chip sizes are under way, and early proficiency-testing schemes mirror those that normalised next-generation sequencing a decade ago. Data-volume bottlenecks are easing as federated learning models that predict volumetric expression from sparse slices reach parity with full-stack 3-D reconstructions, sharply cutting sequencing cost and compute time without sacrificing biologic nuance.

Future research should therefore prioritise multicentre, treatment-naïve and post-treatment spatial cohorts with integrated proteomic and epigenomic layers; exploit AI-guided imputation to democratise 3-D mapping in low-throughput settings; and embed spatial endpoints into adaptive clinical trials so that niche-directed agents can be titrated against objective, voxel-resolved pharmacodynamic read-outs. These advances position 3-D spatial transcriptomics not as a descriptive luxury but as a foundational diagnostic and drug-development engine poised to lift the long-standing therapeutic ceiling in osteosarcoma. Integrating 3-D ST with high-plex protein imaging (IMC or CODEX) enables voxel-level RNA-to-protein validation, refines ligand–receptor maps and reveals post-transcriptional regulation.
